# An Overdose of Chloral during Parturition

**Published:** 1884-09

**Authors:** 


### An Overdose of Chloral During Parturition.

To a young and healthy patient, in her third labor, one hundred grains of chloral hydrate were given, in divided doses, in the course of forty-five minutes, in mistake for three doses of fifteen grains each. The patient became heavy and drowsy, but could be temporarily roused. The pulse was between sixty and seventy per minute. The pains became slow and ineffective. Fifteen minutes after the administration of the last dose of chloral, the membranes were ruptured, and twenty-five minutes later the child was feebly expelled by natural efforts. It was excessively pale, but began to breathe and cry after moderate efforts on the part of the accoucheur, but the skin did not exhibit its natural color for several hours. The placenta soon followed the child. Two drachms of fluid extract of ergot were given, and half an ounce of brandy. An hour after the birth, the mother was in a fairly good condition, and both she and the child made a good recovery.—*Boston Med. and Surg. Jour.*

